# Resveratrol: A potential therapeutic natural polyphenol for neurodegenerative diseases associated with mitochondrial dysfunction

**DOI:** 10.3389/fphar.2022.922232

**Published:** 2022-09-16

**Authors:** Ekta Yadav, Pankajkumar Yadav, Mohd Masih Uzzaman Khan, HariOm Singh, Amita Verma

**Affiliations:** ^1^ Bioorganic and Medicinal Chemistry Research Laboratory, Department of Pharmaceutical Sciences, Sam Higginbottom University of Agriculture, Technology and Sciences, Prayagraj, India; ^2^ Department of Pharmaceutical Sciences, Sam Higginbottom University of Agriculture, Technology and Sciences, Prayagraj, India; ^3^ Department of Pharmaceutical Chemistry and Pharmacognosy, Unaizah College of Pharmacy, Qassim University, Unaizah, Saudi Arabia; ^4^ Department of Molecular Biology, ICMR-National Aids Research Institute, Pune, India

**Keywords:** Alzheimer’s disease, Parkinson’s disease, neurodegenerative diseases, polyphenol, resveratrol

## Abstract

Most polyphenols can cross blood-brain barrier, therefore, they are widely utilized in the treatment of various neurodegenerative diseases (ND). Resveratrol, a natural polyphenol contained in blueberry, grapes, mulberry, etc., is well documented to exhibit potent neuroprotective activity against different ND by mitochondria modulation approach. Mitochondrial function impairment is the most common etiology and pathological process in various neurodegenerative disorders, viz. Alzheimer’s disease, Parkinson’s disease, Huntington’s disease and amyotrophic lateral sclerosis. Nowadays these ND associated with mitochondrial dysfunction have become a major threat to public health as well as health care systems in terms of financial burden. Currently available therapies for ND are limited to symptomatic cures and have inevitable toxic effects. Therefore, there is a strict requirement for a safe and highly effective drug treatment developed from natural compounds. The current review provides updated information about the potential of resveratrol to target mitochondria in the treatment of ND.

## Introduction

Mitochondria are cytoplasmic double-layer organelle, known as a powerhouse of the cell, which plays an important role in many cellular physiological processes including the production of energy by synthesizing adenosine triphosphate (ATP) *via* oxidative phosphorylation (OXPHOS) (de Oliveira et al., 2016). This process involves the transfer of electrons by an electron transport chain (ETC) made up of about 80 polypeptides and forms different trans-membrane protein complexes (I-V) (Dias and Bailly, 2005). Along with energy production, mitochondria aid in several other important functions as discussed below:

### Cellular bioenergetics regulation

Inner membrane of mitochondrial structure consists of four ETC complexes along with ATP synthase leading to the synthesis of ATP molecules (DeBalsi et al., 2017). Two interchangeable and well-regulated process of cell mitochondria, i.e., fusion and fission, causes continuous shape change of mitochondria (van der Bliek et al., 2013). As per metabolic demand, the fission process takes place to increase the mitochondrial count while in the opposite case, the fusion process occurs by the interconnected network (Youle and van der Bliek, 2012). Impairment in the normal physiology of mitochondria such as cellular bioenergetics results in the onset of different NDs (Calkins et al., 2011; Filosto et al., 2011; Fischer et al., 2012). One of the past studies observed the shortage of ATP and faulty mitochondrial OXPHOS in ND patients due to frequent episodes of mitochondrial fragmentation within fibroblast cells (Capaldi et al., 2004). Mitochondrial dysfunction, widely observed in ND, contributes to an elevation in oxidative stress and reduction in ATP synthesis, ultimately affecting the anatomy and physiology of neurons followed by neuronal death (Misrani et al., 2021). Therefore, defective mitochondrial bioenergetics play a vital role in inducing ND-associated pathologies (Bhatti et al., 2022).

### Ca^2+^ homeostasis regulation

Different intracellular signaling pathways including Ca^2+^ homeostasis and apoptosis process are directed by mitochondria (Davis and Williams, 2012). In addition, proper Ca^2+^ homeostasis is further responsible for maintaining the normal structure and function of mitochondria such as pH, membrane potential, energy synthesis and buffering capacity (Johri and Beal, 2012). The Ca^2+^ transport process is maintained by various mitochondrial transport pathways such as Na^+^-dependent Ca^2+^ exchange, Ca^2+^ uniporter and voltage-dependent anion channel (VDAC) (Giorgi et al., 2012; Shoshan-Barmatz et al., 2017). Various neuronal biochemical pathways and activities, i.e., depolarization, plasticity, synaptic activity and neuronal survival, are conserved by intracellular Ca^2+^ (Zündorf and Reiser, 2011; Calì et al., 2012). Overburden of mitochondrial Ca^2+^ level depolarises the mitochondrial membrane by damaging its integrity and increasing oxidative stress leading to a decrease in ATP synthesis, mitochondrial permeability transition pore (mPTP) formation, and neurite growth inhibition. Damage to the neurotransmission process results in neurodegeneration and ultimately onset and progression of ND (Ryu et al., 2010; Zündorf and Reiser, 2011; Calì et al., 2012; Corona and Duchen, 2015).

### Mitochondria in oxidative stress

During the process of energy production *via* OXPHOS, mitochondria simultaneously generate reactive oxygen species (ROS) (superoxide anion and hydroxyl radical) (Federico et al., 2012; Zorov et al., 2014). Additionally, singlet oxygen, reactive nitrogen species (RNS) and hydrogen peroxide (H_2_O_2_) are also formed by mitochondria (Balasaheb Nimse and Pal, 2015; Phaniendra et al., 2015). The balanced amount of mitochondrial ROS/RNS involved in various beneficial processes such as activation of mitogenic response, regulation of various signaling pathways and providing a defense mechanism against pathogens (Sena and Chandel, 2012; Bhat et al., 2015). Overproduction of ROS causes a situation of cellular oxidative stress (Burton and Jauniaux, 2011), which initiates ND early pathogenesis via mitochondrial DNA (mtDNA) damage, lipid peroxidation and protein oxidation associated with mitochondrial dysfunction (Kim et al., 2014; Wang et al., 2014). Neuronal death is observed as a result of peroxidation of cardiolipin (maintains integrity of inner mitochondrial membrane), disruption of cytochrome oxidase activity and disordered ATP generation, induced by oxidative stress (Srinivasan and Avadhani, 2012).

### Mitochondrial dynamics and bioenergetics

Mitochondrial dynamics play a crucial role in retaining the usual mitochondrial functions as well as cell endurance (Suárez-Rivero et al., 2016). Neuronal physiological functions are widely regulated by mitochondrial dynamics and they are highly prone to any modification in mitochondrial dynamics (Gao et al., 2017). Mitochondrial morphology, biogenesis, mitophagy and energy demand are maintained by fission and fusion processes via modulation of expression of concerned genes and proteins through post-translation activity (phosphorylation, ubiquitination and sumoylation) (Suárez-Rivero et al., 2016). Impaired mitochondrial dynamics implicate damaging effects on respiratory chain function, mitochondrial fission and fusion as well as ATP biogenesis leading to neuronal death and onset of NDs pathologies (Burté et al., 2015; Sebastián et al., 2017).

Proliferation of ROS due to depletion of electrons from the ETC is the primary cause of mitochondrial impairment (Rose et al., 2017). However, numerous scientists concerned with mitochondrial dysfunction research have worked with neurodegeneration in neuronal cells that generate energy through OXPHOS and are susceptible to the generation of mitochondrial ROS (Fernandez-Fernandez et al., 2012). Origination and progression of apoptosis as well as cellular ROS generation occur in the mitochondria (Schulz et al., 2014). Neuronal energy demand is fulfilled by OXPHOS in mitochondria, therefore, a shortage of energy due to operational impairment of mitochondria leads to cell death (Rose et al., 2017).

Studies show that mitochondrial dysfunction is the pathological characteristic of neurodegenerative, cardiovascular, gastrointestinal and metabolic disorders as well as cancer. Continuous mitochondrial impairment may lead to detrimental disorders such as disruption of calcium hemostasis in cells, mPTP protein dysfunction and overproduction of free radicals. In recent years, there has been a great preference for biologically active natural substances in the amelioration of mitochondrial activity (Fiorani et al., 2010; Chen et al., 2012; Abbaszadeh et al., 2014). Among various natural bioactive materials, polyphenols such as resveratrol (RES) have been reported to be useful substances for the improvement of mitochondrial functions (Davinelli et al., 2013; Ferretta et al., 2014; de Oliveira et al., 2016). Extensive research has been performed on the role of RES in various ND. The current review highlights updated knowledge about the ability of RES to target mitochondria in ND treatment.

## History and background of RES

**FIGURE 1:**
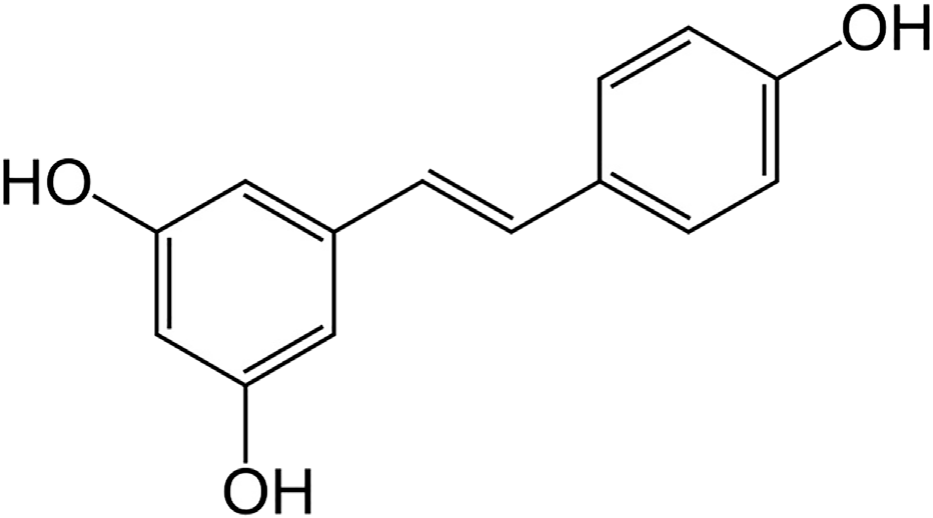
Structure of RES.

RES (Figure 1) is a polyphenol obtained from the phenylalanine/polymalonate pathway in plants (Jeandet et al., 2020, 2021). It is a stilbene (molecules of two phenolic rings linked by ethene) polyphenolic compound. RES was first time isolated in the year 1940 from roots of the plant Veratrum grandiflorum (*white hellebore*), and later on from Polygonum cuspidatum (*Asian knotweed*) roots. Initially, RES was recommended for cardiovascular disorders. To date, researchers have reported that RES is an effective agent in the treatment of cancer, pain, inflammation, wound repair and associated conditions (Tellone et al., 2015).

RES has gained the attention of scientists in recent years to investigate its effects on aging and neurodegenerative diseases. Several types of interaction with receptors have been proposed to explain the neuroprotective effect of RES, however, its antioxidant ability is the most promising property against the pathologies associated with neuronal impairment. Numerous researchers have advocated the therapeutic potential of RES in many neurodegenerative disorders [i. e., Parkinson’s disease (PD), Alzheimer’s disease (AD), Huntington’s disease (HD) and amyotrophic lateral sclerosis (ALS)] (de Oliveira et al., 2016).

Favorable qualities of red wine are already reported in prehistorical literature. Ayurveda, the ancient text of Hindus, has mentioned the cardiotonic properties of fermented red grape juice (Paul et al., 1999). According to Holy Bible, Timothy, a Christian devotee, was permitted to drink a small quantity of wine for his recurrent digestive disorders. Firstly, RES was designated as a phytoalexin (a substance produced by plants in reaction to infection or damage) from leaf epidermis and grape skin of *Vitis vinifera* (common grape vine) (Langcake and Pryce, 1976).

The interest of scientists in research on RES was limited for approximately 50 years after its discovery. A research paper suggesting ‘RES as a lipid-lowering substance in wine’ attracted the attention of researchers in 1992. Later on, several scientists performed the research on beneficial properties of RES on the circulatory system and proposed that consumption of RES is one of the causes of the low prevalence of cardiovascular disorders in France, where people consume a decent amount of wine with high-fat food (Renaud and de Lorgeril, 1992; Goldberg et al., 1995). In 1997, researchers suggested RES as a chemopreventive agent (Jang et al., 1997). Thereafter, RES has remained in the focus of scientists and many of its activities have been reported (e.g., antioxidant, anti-ischemic, analgesic, anti-inflammatory) (Frémont, 2000; Granados-Soto, 2003; Baur and Sinclair, 2006). In recent years, the scientific investigation on RES is centered on its role in neurodegenerative diseases and the evaluation of responsible mechanisms.

### Chemistry of RES

Chemically RES is 3,5,4′-trihydroxystilbene and it is a stilbene family member (Keylor et al., 2015). It is further identified as a phytoalexin (Zhou et al., 2021). The reaction between p-coumaroyl-coenzyme A and three malonyl-coenzyme A molecules is accomplished by RES synthase (modulated by defense system of the plant) in shikimic acid pathway to form RES (Ibrahim et al., 2021). Cis and trans structure of RES, free or attached to a glucose molecule, has been obtained. At first, cis-resveratrol was believed as a non-bioactive substance. Later on, many reports showed that both the isomers were biologically active, however, trans-isomer was observed to be more effective in various comparative studies, indicative of stereo-specific biological activity of RES (Salehi et al., 2018). It is a white-colored powder, very slightly soluble in warm water and freely soluble in dimethyl sulfoxide as well as ethanol. The trans form of RES has better stability in comparison to cis configuration (Salehi et al., 2018). Even in extracts, trans-RES is usually found in a bound state with other molecules, such as sugars. It dimerizes to cis isomer by exposure to light (Kosović et al., 2020). Trans-RES degrades exponentially in alkaline pH, while it was stable in acidic pH at body and room temperature (Robinson et al., 2015; Zupančič et al., 2015). It is highly bound with plasma proteins. Degradation of RES was recorded by first-order kinetics with 54 h and 25 h half-lives in human and rat plasma, respectively (Robinson et al., 2015).

### Sources of RES

RES is found in a wide variety of plants, which are not related taxonomically (Table 1). Edible plants such as cocoa, grapes, tomato fruit skin, peanuts, blueberries, jackfruit, bilberries, and mulberries have variable amounts of RES (Stewart et al., 2003; Hurst et al., 2008). Moreover, the level of RES varies in different conditions of the same fruit or plant. For example, the amount of RES in ripening grapes increases following UV exposure or bacterial and fungal infection. Additionally, a higher amount of RES is present in grapes grown in cold weather. A study suggests that cocoa products have less quantity of RES in comparison to grape juice and red wine (Hurst et al., 2008). Different biological activities are exhibited by plant families such as Cyperaceae, Vitaceae, Dipterocarpaceae and Gnetaceae, which contain RES oligomers (Ito, 2020).

**TABLE 1:** Sources of RES.

Plant common name	Biological source	Family	Plant part	References
Peanut	*Arachis hypogea*	Fabaceae	Root	Chen et al. (2002)
Jackfruit	*Artocarpus heterophyllus*	Moraceae	Fruit skin	Shrikanta et al. (2015)
Grapes	*Vitis venifera*	Vitaceae	Fruit skin, pulp and seed	Shrikanta et al. (2015)
Blueberry	*Vaccinium myrtillus*	Ericaceae	Fruit	Lyons et al. (2003)
Mulberry	*Morus alba*	Moraceae	Fruit	Shrikanta et al. (2015)
European cranberry	*Vaccinium oxycoccus*	Ericaceae	Fruit	Jurikova et al. (2018)
White hellebore	*Veratrum grandiflorum *	Melanthiaceae	Root	Salehi et al. (2018)
Corn lily	*Veratrum lobelianum*	Melanthiaceae		Dirks et al. (2021)
Scots pines	*Pinus sylvestris*	Pinaceae	Whole plant extract	Ferrante et al. (2020)
Hops	*Humulus lupulus L*	Cannabaceae		Callemien et al. (2005)
Rhapontic rhubarb	*Rheum rhaponticum*	Polygonaceae	Roots	Kolodziejczyk-Czepas and Liudvytska, (2021)
Garjan	*Dipterocarpus grandifloras*	Dipterocarpaceae	Stem bark	(Ito et al., 2004, 2009)
Joint Fir	*Gnetum montanum*	Gnetaceae	Roots	Shen et al. (2017)
Japanese knotweed	*Polygonum cuspidatum*	Polygonaceae	Roots	Ghanim et al. (2010)
Strawberry	*Fragaria ananassa*	Rosaceae	Fruit	Wang et al. (2007)
Penyau	*Upuna borneensis*	Dipterocarpaceae	Stem	Ito et al. (2005)
Tomato	*Lycopersicon esculentum*	Solanaceae	Fruit skin	Ragab et al. (2006)

### Bioavailability and toxicity studies of RES

Different routes of administration and doses of RES have been used by researchers in their studies involving rats, mice and human models. Therefore, it is challenging to interpret the functional dose of RES in humans concerning various diseases. It is important to fix a range of dose to consider RES in the effective prevention, management or treatment of a disease. It has been reported that 3 g per kg dose of RES induced oxidative stress along with nephrotoxicity, DNA damage and apoptosis in rats (Salehi et al., 2018; Shaito et al., 2020). Translation of such a large dose for a human may result in a very high equivalent dose (approx. 33.87 g per day for an adult weighing 70 kg) (Nair and Jacob, 2016). Therefore, it is important to cogitate the side effects due to high dose when the efficacy of RES in the brain is considered. RES possesses poor pharmacokinetic properties as it has low solubility and low bioavailability (25 mg of oral dose produces <5 ng/ml of unmetabolized RES in plasma) (Yang et al., 2021). Conversely, RES has been reported to possess the property of crossing the BBB (0.0456 mg per kg) (Shu et al., 2015). Most of the researchers have administered 100–200 mg/kg dose of RES in animals through oral route and reported no side effects along with substantial alterations in neuronal transmission. Administration of 0.3 g per kg per day dose to humans (weight 70 kg) revealed no renal or hepatic toxicity (Crowell et al., 2004). Generally, effects of RES on the brain were examined at various doses in animal models and justified amounts of doses were administered in humans for the same. Minor side effects (nausea, diarrhea, headache) were exhibited by administration of RES in acceptable dose level, up to 5 g per day, revealing that a very large dose is required to initiate toxic effects (Salehi et al., 2018; Shaito et al., 2020). Rahman et al., 2020 and Ramírez-Garza et al., 2018 have reported more information about RES dosage and tolerance in animals as well as human models.

Metabolic products of RES exhibited micromolar concentration in plasma (Corder et al., 2003; Kaldas et al., 2003; Walle et al., 2004). A study showed that glucuronidation is the primary mechanism of RES metabolism (Kuhnle et al., 2000). A researcher identified trans-resveratrol-3-O-glucuronide and trans-resveratrol-3-sulfate in mouse serum and rat urine along with nominal quantities of intact RES (Yu et al., 2002). Another study reported that trans-resveratrol-3,4′-disulfate, trans-resveratrol-3-O-beta-D-glucuronide, trans-resveratrol-3,5-disulfate, trans-resveratrol-3- sulfate, trans-resveratrol-4′-sulfate, trans-resveratrol-3,4′,5-trisulfate were detected as biotransformation products in the animals subjected to fast metabolism (Corder et al., 2003; Walle et al., 2004). Studies on humans exhibited analogous outcomes revealing trans-resveratrol-3-sulfate and trans-resveratrol-3-O-glucuronide as primary metabolites (De Santi et al., 2000a; De Santi et al., 2000b). These metabolites are eliminated by ATP binding cassette (ABC) membrane proteins (Planas et al., 2012). Thus, RES metabolism in the intestine is postulated to be the primary reason for its lower absorption. Hydroxyl groups of RES are primarily conjugated with glucuronide and sulfate, whereas acetylated hydroxyl groups are resistant to glucuronidation imparting more stability and improved pharmacokinetic characteristics (Liang et al., 2013). Superior hydrophobic nature of acetylated RES enhanced the permeability through cell membrane as compared to the parent molecule (Lançon et al., 2004). Researchers revealed that ABC transporters (e.g., breast cancer resistance protein, BCRP, and multidrug resistance associated protein 2, MRP2) are responsible for elimination of trans-RES metabolic products into the intestinal lumen. These transporter proteins restrict the absorption of tans-RES and are possibly accountable for distribution of RES metabolites in various tissues (Planas et al., 2012). Concentration of RES was recorded in different organs such as brain, lung, kidney, heart, spleen, liver, small intestine and stomach after administration. Lungs and heart exhibited the maximum concentration of the compound. Initially, the concentration of RES increased in the tissues after administration and it was decreased later on. The study results exhibited that RES distribution is dependent on blood perfusion rate in respective organs (Liang et al., 2013). In spite of low oral bioavailability, a number of beneficial effects of RES have been suggested through various mechanisms via *in vivo* studies on animals and humans. It is demonstrated that enterohepatic circulation is the primary mechanism of reabsorption RES and its metabolites, which contributes to their overall bioavailability (Marier et al., 2002). Some researchers have discovered that metabolic products of RES have therapeutic potential in different animal models. A study on chemopreventive potential of RES (orally administered) and its sulphate metabolites showed quinone reductase 1 (QR1) induction, antioxidant activity and cyclooxygenase-1 (COX-1) and cyclooxygenase-2 (COX-2) inhibition. The researchers justified that a higher plasma concentration of sulfate metabolites in comparison to RES could be responsible for the biological activity. Another research advocated that COX-1 and COX-2 were inhibited by RES and its sulfate metabolite (resveratrol 4′-O-sulfate). Additionally, RES could downregulate quinone reductase 2 (QR2). The authors concluded that RES and its metabolites have diverse target sites and this property might be accountable for health benefits of RES (Hoshino et al., 2010).

## Role of mitochondrial dysfunction in various NDs

Defect in mitochondrial function is considered a significant pathological hallmark in various ND attributed to OXPHOS linked reduction in ATP synthesis, depolarization of the mitochondrial membrane, imbalance in Ca^2+^ homeostasis, damaged mitochondrial fission/fusion process, mtDNA destruction, oxidative stress, stimulation of genes concerned with pro-apoptotic, etc. (Harris et al., 2014). Lower quality control of mitochondria has been suggested by various reports associated with different ND including PD, AD, ALS and HD, etc. (Wager and Russell, 2013; Ni et al., 2015), still the responsible molecular mechanism for ND is not clear. Impaired mitochondrial function results in deficiency of ATP and neuronal death followed by the onset of ND. Mitochondria are an exclusive source of energy production, therefore, researchers are focusing on the investigation of ND pathologies induced by mitochondrial damage (Federico et al., 2012). A new therapeutic approach, against mitochondria impairment associated ND, can be explored by targeting the novel mechanisms involved in the quality control of mitochondria along with neuronal health.

### Mitochondrial dysfunction and AD

The population of aged people is continuously growing at a faster rate all over the world and due to this, age-related disorders including AD are present as a severe social and economic issue (Ballard et al., 2011). Amyloid-β (Aβ) deposition, neurofibrillary tangles (NFTs) and hyperphosphorylation of tau protein are the main causative factors in AD. Mitochondria are the specific oxidative metabolism organelle in eukaryotic cells. The process of oxidative metabolism produces ROS as their byproducts, which contributes to mitochondrial dysfunction such as poor mitochondrial biogenesis, mtDNA mutations and deficit in mitochondrial dynamics (Yun and Finkel, 2014). Persistent ROS-induced neuronal mitochondrial dysfunction can lead to increased production and aggregation of Aβ as well as hyperphosphorylation of tau by activation of glycogen synthase kinase-3 (GSK-3). Regarding AD, mitochondrial dysfunction promotes the level of oxidative stress which leads to the formation of 4-hydroxynonenal (lipid peroxidation product) followed by Aβ accumulation via modulation in γ-secretase complex as well as secretase activity (Chen et al., 2015). A preclinical study observed the reversal in the aggregation of tau protein induced by a reduced level of mitochondrial superoxide dismutase 2 (SOD2) in mice model by antioxidant treatment (Melov et al., 2007). Moreover, the anatomical integrity and physiology of mitochondria are damaged by NFTs and cause the onset and further progression of AD (Kerr et al., 2017). Neuronal mitochondrial dysfunction persuaded by ROS may act as an essential factor in sporadic AD pathogenesis (Swerdlow and Khan, 2004). Therefore, utilization of potential drug agents against ROS-induced oxidative stress to provide mitochondrial protection might be a promising therapeutic tool for the treatment of sporadic AD (Hardy and Allsop, 1991). Recently, Sorrentino et al., 2017 suggested that reduction in cellular Aβ aggregation can be achieved by targeting the mitophagy process via pharmacological and genetic means. RES possesses a broad spectrum of therapeutic effects such as antioxidant, antiaging, neuroprotection and antiAD (Sawda et al., 2017).

It has been suggested that Aβ42 plaques accumulation was significantly endorsed by mitochondrial oxidative stress via altering amyloid precursor protein-like (APPL) protein expression by following the amyloidogenic pathway (Zuo et al., 2015). Moreover, it also influenced several other signaling pathways associated with cellular stress marker, c-Jun N-terminal kinase (JNK) as well as nuclear factor kappa-light-chain-enhancer of activated B cells (NF-κB) (Anfinogenova et al., 2020). Modifications in mitochondrial dynamics induce a damaging effect on cells and may further cause the initiation and progression of AD (Sheng et al., 2012; Golpich et al., 2017). An elevated level of oxidative stress contributes to mitochondrial dynamic impairment and reduction in guanosine triphosphate hydrolase (GTPase), i.e., dynamin like protein 1 (DLP1), an expression that is known to control mitochondrial fission and ultimately promotes Aβ generation in AD (Akbar et al., 2016). In continuation, mitochondrial autophagy also contributes to sustaining neuronal integrity but still, the mechanism of AD and autophagy is not well known (Kamat et al., 2014; Martinez-Vicente, 2017). Pathological study of patient brain suffering from AD exhibited the aggregation of autophagosomes as well as pre-lysosomal autophagic vacuoles that further caused the accumulation of Aβ plaques (Nixon et al., 2005; Yu et al., 2005). It has been demonstrated that a deficit in the respiratory chain in response to mitochondrial structural change promotes the overgeneration of ROS and reduction in ATP concentration which results in lack of energy, neuronal death and NDs (Readnower et al., 2011). Moreover, mitochondrial dysfunction is induced by Aβ toxicity via modulating the mitochondrial fission and fusion process.

### Mitochondrial dysfunction and PD

PD is a progressive neurodegenerative disorder characterized by tremors, increase in muscle rigidity, bradykinesia and almost complete loss of movements in extreme cases. Symptoms associated with motor function take place due to dopaminergic neurodegeneration of the substantia nigra leading to deficiency in dopamine and intracytoplasmic Lewy bodies aggregation consisting *α*-synuclein and ubiquitin (Blesa et al., 2012). Monoamine oxidase (MAO) enzyme plays a vital role in the inactivation of dopamine and subsequently produces a huge amount of hydrogen peroxide that is required to be detoxified continuously by intracellular antioxidants defense system. In PD, the basic pathway for dopaminergic cell death is apoptosis instead of necrosis (He et al., 2000). Oxidative stress as well as nitrative stress in substantia nigra are considered potential contributors to the onset of PD (Giasson et al., 2002). The causative factor of oxygen radical origin is still not well known but secondarily related to several biochemical alterations including an imbalance in antioxidant defense mechanism as well as iron homeostasis and dysfunction in mitochondria. While the origin of nitrogen species is directly based on modification in iNOS activity. Numerous past reports evidenced the key role of mitochondrial impairment in the initiation of PD pathogenesis. Free radicals are majorly produced by inhibition of Complex I and modulation of its function can cause decreased ATP production as well as increased oxidative stress, which amplifies the origination of neurodegenerative disease (Bhatti et al., 2017).

### Mitochondrial dysfunction and HD

HD is concerned with neurodegeneration induced by alteration in mutant HTT gene due to expansion of CAG repeat process results in size elevation of polyglutamine (polyQ) tract within the *N*-terminal of the Huntington (Htt) protein. The mutant Htt (mHtt) protein along with extended polyQ starts to aggregate and damage the process of mitochondrial fission-fusion as well as mitochondrial transportation by directing other related proteins (Reddy and Shirendeb, 2012). Destabilization and increase in Ca^2+^ susceptibility of mitochondrial membrane directly interfered by mHtt (Choo et al., 2004). Overproduction of ROS leads to mitochondrial dysfunction, especially, an increase in the fragmentation process along with a reduction in motility and respiration in patients with HD and rodent models (Gil-Mohapel et al., 2014). Glyceraldehyde 3-phosphate dehydrogenase (GAPDH) plays a vital role in the glycolytic pathway. Normally, the process of engulfing injured mitochondrion by lysosomes is introduced by oxidized inactive GAPDH. Past studies revealed that modulation in GADPH mitophagy was observed in HD cell models, which was possibly mediated by an atypical interface between expanded polyglutamine repeats and GAPDH (Hwang et al., 2015).

The results collected from different studies from mice, cell lines, primary neurons as well patients suffering from HD advised diverse features related to mitochondrial dysfunction, especially affecting the striatum. Early-stage mitochondrial impairment involves the destruction of Ca^2+^ homeostasis, ATP synthesis and transportation process, while respiratory chain impairment occurs at secondary level. Underlying mechanism of mitochondrial dysfunction comprises direct and indirect mHtt pathogenesis. Direct mHtt-mitochondria interactions have the ability to produce an absurdly selective pattern of neurodegeneration in HD. Direct or indirect (through modulation of transcriptional process) effect of mHtt on nuclear-encoded mitochondrial anatomy, preferably in striatal neurons, is due to an exclusive pattern of gene expression, which primarily affects the selectivity process. It has been suggested that a deficit in mitochondrial trafficking within striatal neurons is endorsed by soluble mHtt. Whereas aggregate mHtt affects the cortical neurons, which are possibly less sensitive. Although, relatively more axonal length of striatal neurons as compared to cortical neurons makes them more susceptible to mitochondrial trafficking damage. Nevertheless, this susceptibility is not directly concerned with the length of axon; otherwise, episodes of neuron degeneration in long motor neuron occurs more frequently in HD as compared to striatal degeneration (von Lewinski and Keller, 2005). Furthermore, recent reports evidenced that mitochondrial dysfunction instigated by mHtt primarily contributes to the stimulation of innate and multifactorial striatal vulnerability in place of striatal neurodegeneration in HD. Sirtuin 1 (SIRT1) is nicotinamide adenine dinucleotide dependent lysine deacetylase responsible for increased metabolism of mitochondria and regulates permanence (Naia et al., 2017).

### Mitochondrial dysfunction and ALS

ALS is a progressive neurological disease that primarily affects the brain as well as spinal cord associated motor neurons. Above 90% cases of ALS are sporadic in nature, characterized by abnormalities in movements and damage to intellectual functions. ALS disease mechanisms promisingly involve the process of mitophagy (removal of impaired mitochondria). A preclinical study showed a reduced number of phagosomes in the neuromuscular junction of SOD1^G39A^ mice model as compared to wild-type mice, which revealed interruption in mitophagy process. Additionally, phosphatase and tensin homolog-induced putative kinase 1 (PINK1) and Parkin proteins associated with mitophagy are also found in modulated form. ALS similar symptoms such as aggravation in neuromuscular junction degeneration along with axon swelling were observed in PINK1-Parkin double-knockout mice model. Moreover, elevated levels of ATP synthase beta subunit were also detected in double-knockout mice, which is known to be attributed to damaged mitophagy (Rogers et al., 2017).

## Therapeutic approaches of RES in different NDs

A brief outcome of RES in mitochondrial dysfunction associated with ND is discussed below. Clinical trials of RES on various ND are given in Table 2.

**TABLE 2:** Clinical trials of RES on neurodegenerative diseases

Study title	Phase	Study design	Date of completion	Purpose	Dose	Enrolment	Results	Reference
RES and Huntington Disease	Not applicable	Randomized, double-blind, parallel, placebo-controlled study	January 2020	Measurement of the rate of caudate atrophy before and after 1 year of treatment with RES in early affected HD patients using volumetric MRI.	40 mg of RES orally twice daily	102 early affected HD patients	Not posted	NCT02336633
RES for Alzheimer’s Disease	2	Double blind, placebo-controlled, parallel design	March 2014	Evaluation of biomarkers in RES treated patients with mild to moderate AD	500 mg once daily increasing at 13 weeks intervals to a maximum of 1 g twice daily (oral)	120 patients with mild to moderate dementia due to probable Alzheimer’s disease	RES and its major metabolites penetrated BBB. CSF Aβ40 and plasma Aβ40 levels declined significantly in placebo group. Brain volume loss was increased by RES Turner et al., (2015); Moussa et al., (2017)	NCT01504854
Randomized Trial of a Nutritional Supplement in Alzheimer’s Disease	3	Single Center, Multi-site Randomized, Double-blind, Placebo-controlled Parallel Trial	June 2011	To measure various scores on AD scale in subjects with AD administered with dextrose, malate, and RES.	15 ml of the following preparation per dose, i.e., 5 g dextrose, 5 g malate, and 5 mg RES, or matching placebo with an 8 oz glass of commercial unsweetened grape juice twice a day	39 individuals of 50–90 years of age with mild to moderate AD who were free of life-threatening disease	Low dose of oral RES is well tolerated. Various scores of AD scale showed less degeneration in the treatment group, however, the change was not statistically significant Zhu et al., (2018)	NCT00678431
Effect of Food on BIA 6-512 (Trans-RES)	1	Single-center, open-label, randomized, two-way crossover study	July 2005	To determine the effect of Food on the Pharmacokinetics of a Single 400 mg Oral Dose of BIA 6-512 (Trans-RES) in Healthy Subjects	400 mg of trans-RES administered orally in fed and fasted groups	24 healthy male and female subjects	Not posted	NCT03095092
Tolerability and Steady-state Pharmacokinetics of BIA 6-512 (Trans-RES)	1	Double-blind, Randomized, Placebo-controlled, Rising Multiple-dose Study	July 2005	To investigate the tolerability and safety of four multiple-dose regimens and characterize the steady-state pharmacokinetic profiles of BIA 6-512 in healthy volunteers.	25 mg, 50 mg, 100 mg, and 150 mg of trans-RES, 6 times daily, oral	40 healthy volunteers	Not posted	NCT03093389
Pharmacokinetic Profile of BIA 6-512 in Healthy Elderly Subjects Versus Healthy Young Subjects	1	Single-center, Open-label, Parallel-group Study	March 2006	To compare pharmacokinetic profile of BIA 6-512 in healthy elderly subjects versus healthy young subjects after single and repeated oral administration of 200 mg BIA 6-512.	200 mg BIA 6–512 oral dose on Day 1, 200 mg thrice on day 2 and day 3, and 200 mg on day 4 in young (18–40 years) as well as elderly (65 years or above) groups	25 healthy subjects	Not posted	NCT03095105
Impact of the Combined Treatment of Liposomed Polyphenols With G04CB02 (dutasteride) on amyotrophic lateral sclerosis (ALS) Patients	2	Double-blind, Randomized, Placebo-controlled, Parallel-group Study	Study is active	To study the impact of the combined treatment of curcumin and RES liposomed polyphenols with G04CB02 (dutasteride) on the clinical improvement of ALS patients	Combination of RES (75 mg) and curcumin (200 mg) liposomes with dutasteride (0.5 mg), in a single dose	60 patients with Amyotrophic lateral sclerosis (ALS)	Study is active, Recruiting	NCT04654689
Bioactive Dietary Polyphenol Preparation (BDPP) Treatment for Mild Cognitive Impairment and Prediabetes or Type 2 Diabetes Mellitus	1	Double-blind, Randomized, Parallel Study	Study is active	To measure penetrance of BDPP in CSF, assessment of adverse events and evaluation of scale of dementia.	Low, moderate and high doses of grape seed polyphenolic extract, and RES	48 patients with amnestic Mild Cognitive Impairment and Type 2 diabetes	Study is active, Recruiting	NCT02502253
Tolerability, Safety and Pharmacokinetics of Four Single-doses of BIA 6-512 (Trans-RES) and Their Effect on the Levodopa Pharmacokinetics	1	Single-center, double-blind, randomized, placebo-controlled, crossover study	July 2004	To investigate the effect, tolerability and safety of four single oral doses of BIA 6-512 on levodopa pharmacokinetics.	25 mg, 50 mg, 100 mg and 200 mg of trans-RES and 100/25 mg of levodopa/benserazide	20 healthy subjects	Not posted	NCT03091543
Therapeutic Metabolic Intervention in Patients With Spastic Paraplegia SPG5 (SPA-M)	2	Open-label, randomized, crossover study	September 2017	To study the efficacy of three candidate molecules (Xenbilox, Tahor and RES) in order to decrease the production of oxysterols by reducing the synthesis of cholesterol and/or regulate the production of bile acids and/or enabling neuroprotective action within the motor neuron.	80 mg capsule of RES, Xenbilox or Tahor by mouth	12 patients that have confirmed through genetic testing their status as carriers of 2 mutations in the CYP7B1 gene	Not posted	NCT02314208
Effect of BIA 6-512 at Steady-state on the Levodopa Pharmacokinetics	1	Single-center, Double-blind, Randomized, Placebo-controlled, Rising Multiple Dose Study	July 2006	To Investigate the Effect of BIA 6-512 at Steady-state on the Levodopa Pharmacokinetics When Administered in Combination With a Single-dose of Levodopa/Benserazide or With a Single-dose of Levodopa/Benserazide Plus a Single-dose of Entacapone	25 mg, 50 mg, 75 mg and 100 mg of RES with a Single-dose of Levodopa/Benserazide 200/50 mg or With a Single-dose of Levodopa/Benserazide 200/50 mg Plus a Single-dose of Entacapone 200 mg	20 healthy subjects	Not posted	NCT03094156
Effect of BIA 6-512 at Steady-state on the Levodopa Pharmacokinetics With a Single-dose of Levodopa/Benserazide 200/50 mg or With a Single-dose of Levodopa/Benserazide 200/50 mg Plus a Single-dose of Nebicapone 150 mg	1	Single-center, double-blind, randomized, placebo-controlled, rising multiple-dose study	October 2006	To determine whether the administration of BIA 6–512 at steady-state affects the pharmacokinetics of levodopa when administered in combination with a single dose of immediate-release levodopa/benserazide or with a single-dose of immediate-release levodopa/benserazide plus a single-dose of nebicapone.	25 mg, 50 mg, 75 mg and 100 mg of RES with a Single-dose of Levodopa/Benserazide 200/50 mg or With a Single-dose of Levodopa/Benserazide 200/50 mg Plus a Single-dose of Nebicapone 150 mg	38 healthy subjects	Not posted	NCT03097211
Short Term Efficacy and Safety of Perispinal Administration of Etanercept in Mild to Moderate AD	1	Randomized, Open Label, Crossover Study	October 2015	To Assess the Efficacy & Safety of Perispinal Administration of Etanercept (Enbrel^®^) in Combination with Nutritional Supplements or Alone in Subjects with Mild to Moderate AD.	Curcumin, Luteol, Theaflavins, Lipoic Acid, Fish Oil, Quercetin and RES with or without Etanercept	12 patients of AD	Not posted	NCT01716637
Pharmacokinetics of Rising Single-doses of BIA 6-512 and Their Effect on the Levodopa Pharmacokinetics	1	Single-center, double-blind, randomized, placebo-controlled study	February 2005	To investigate the effect of rising oral single-doses of BIA 6-512 (25 mg, 50 mg, 100 mg and 200 mg) on levodopa pharmacokinetics when administered in combination with a single-dose of immediate release levodopa/carbidopa 100/25 mg (Sinemet^®^ 100/25) or with a single-dose of Sinemet^®^ 100/25 plus a single-dose of entacapone (Comtan^®^) 200 mg.	Oral single-doses of BIA 6-512 (25 mg, 50 mg, 100 mg and 200 mg) with a single-dose of immediate release levodopa/carbidopa 100/25 mg (Sinemet^®^ 100/25) or with a single-dose of Sinemet^®^ 100/25 plus a single-dose of entacapone (Comtan^®^) 200	80 healthy subjects	Not posted	NCT03091868

Source: https://www.clinicaltrials.gov/

### RES in AD

**FIGURE 2:**
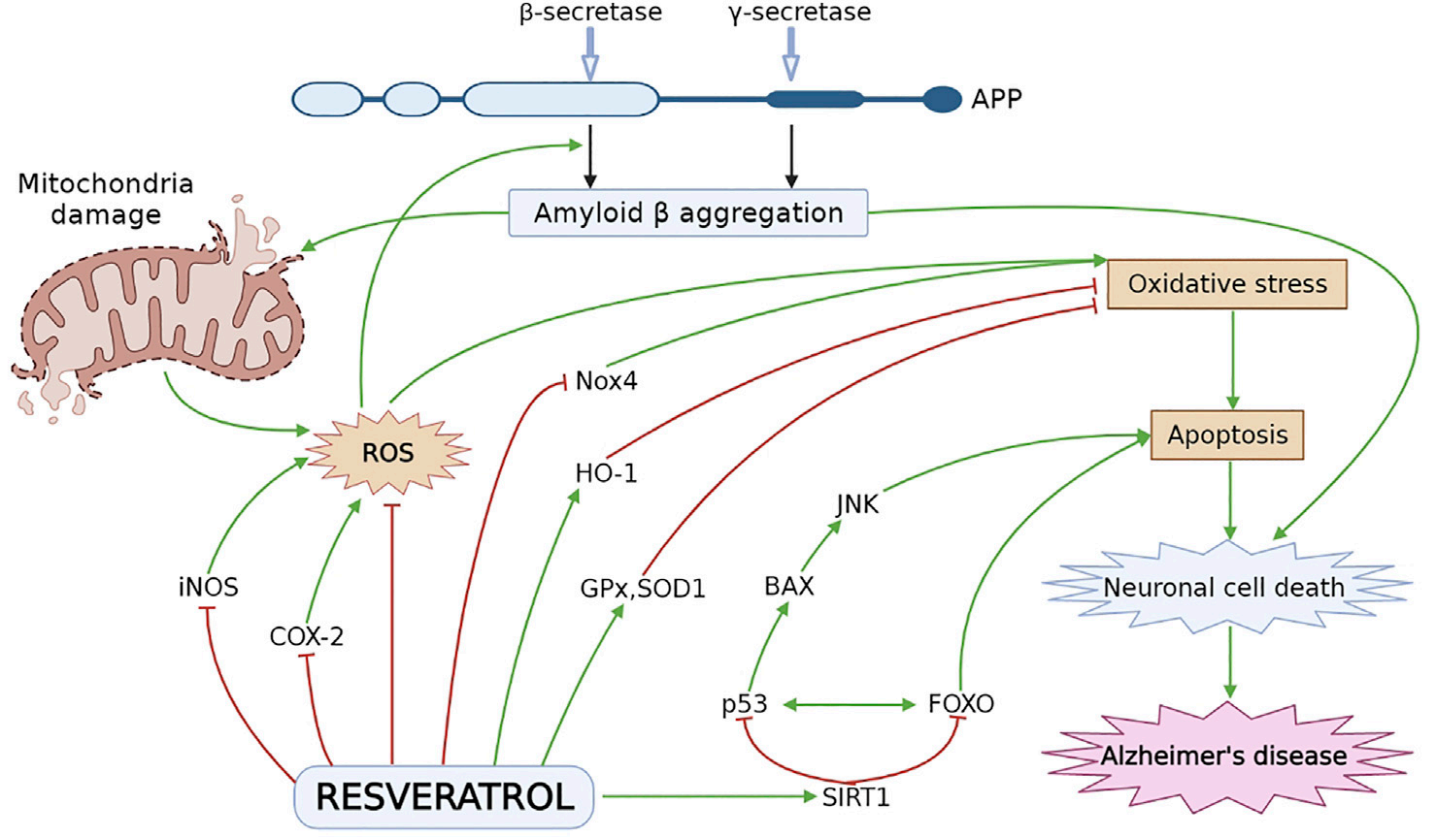
Neuroprotective effect of RES in AD pathogenesis. Oxidative stress leads to formation of ROS especially, iNOS and COX-2, which plays a vital role in cellular apoptosis. RES decreases the level of iNOS and COX-2 and stimulate the HO-1 level to inhibit oxidative damage. RES reduces oxidative stress by decreasing the Nox4 expression and elevating the level of ROS inactivating enzymes, i.e., SOD1 and GPx. RES activates SIRT1 followed by inhibition of p53 and FOXO to attenuate BAX and JNK dependent apoptosis and neuronal cell death. APP, amyloid precursor protein; BAX, Bcl-2-associated X protein; COX-2, cyclooxygenase-2; HO-1, heme oxygenase-1; iNOS, inducing nitric oxide synthase; JNK, Jun N-terminal kinases; Nox4, NADPH oxidase 4; ROS, reactive oxygen species; SIRT1, sirtuin 1.

Numerous researchers explored the neuroprotective effect of RES by using various *in vitro* and *in vivo* experimental models associated with AD (Feng et al., 2013; Porquet et al., 2014; Freyssin et al., 2020; Rao et al., 2020). AD-induced pathology is significantly improved by RES via modulating different underlying mechanisms and signaling pathways, which can slow down the initiation and further progression of AD Figure 2 (Ahmed et al., 2017; Sawda et al., 2017). As it has been noticed that overproduction of ROS caused by oxidative stress ultimately affects metal homeostasis, mitochondrial activity, antioxidant defense system as well as synaptic function and induces AD-linked neuronal damage. Against this pathology, an antioxidant agent such as RES may be utilized for the treatment and management of AD (Chen and Zhong, 2014; Tönnies and Trushina, 2017). Different *in vitro* and *in vivo* studies have revealed the protective effect of RES in Aβ-induced neuronal oxidative damage (Rahman et al., 2020). RES increases the level of intracellular antioxidants, i.e., glutathione (Kwon et al., 2010) as well as antioxidant enzymes including superoxide dismutase, glutathione peroxidase and catalase (CAT) (Chiang et al., 2018; Lin et al., 2018; Zhao et al., 2018; Kong et al., 2019), and reduce lipid peroxidation (Sharma and Gupta, 2002; Kong et al., 2019). Furthermore, RES protects the disrupted mitochondrial membrane and decreases ROS generation in brain tissue (Kwon et al., 2010).

Pretreatment with RES induced the elevation in gene expression associated with the antioxidant defense system and SIRT-1 in lymphoblastoid cell lines in patients with AD (Cosín-Tomàs et al., 2019). It has been reported that mitochondrial impairment is widely attributed to neurodegeneration and AD because dependence on OXPHOS continuously increases with aging (Harris et al., 2014). Further metabolic dysregulation occurs as a result of elevation in OXPHOS as well as a decrease in aerobic glycolysis via an increase in ROS level (Harris et al., 2014). A study showed that RES has the protective ability to improve the health of mitochondria by modulating peroxisome proliferator-activated receptor coactivator-1α (PGC-1α) activation and expression which is responsible for mitochondrial biogenesis (Scarpulla, 2011), and defending it from metabolic dysregulation through a decrease in superoxide radicals synthesis by stimulating complex III activity (Zhao et al., 2018). Moreover, PGC-1α activation is also attributed to adenosine monophosphate-activated protein kinase (AMPK). Metabolic processes, as well as energy homeostasis, are majorly regulated by AMPK and upon activation, it causes the transcription of various proteins such as PGC-1α, which is required for metabolic regulation (Price et al., 2012). Mitochondrial biogenesis is induced by SIRT1 by involving AMPK. A preclinical study on mice without providing any treatment demonstrated that overexpression of SIRT1 results in an increase in AMPK activity in skeletal muscle, while on the other hand, elevation in AMPK activity was not observed in SIRT- KO mice pretreated with RES at two dose levels of 25 and 215 mg/kg/day. Furthermore, it has also been noted that RV did not induce any significant acceleration in mtDNA content, mitochondrial respiration and PGC-1α level in SIRT1 deficient mice but all parameters were noticed in SIRT1-KI mice (Price et al., 2012). This showed regulation of mitochondrial health, as well as its physiology, are induced by SIRT1 and it is dependent on AMPK. Therefore, RES’ neuroprotective effect is mediated by targeting SIRT1.

Other findings also showed that SIRT1 activity is promoted by RES through stabilization of protein-substrate interactions (Lin C.-H. et al., 2014). RES is observed to make alterations at SIRT1 N-terminal domain confirmation which induces the strong binding between SIRT1 and substrate (Yan et al., 2020). Level of SIRT1 mRNA, as well as protein expression, were also accelerated by RES treatment (Kim et al., 2018; Yan et al., 2020). RES also protects the functioning of SIRT1 from different damaging effects and can act as a SIRT1 agonist (Hu et al., 2016; Yan et al., 2020). RES is believed to induce inhibition of inflammatory response, decrease oxidative stress and apoptosis, and enhance autophagic flux normalization via SIRT1 signaling pathway in human-derived neuroblastoma cell lines (Hou et al., 2016; Zhang et al., 2017). It has been demonstrated that autophagy majorly contributes to the clearance of aggregate-prone proteins which is linked with different ND, including AD (Meng et al., 2015). Neuroprotective effect of RES was observed in PC12 Aβ25-35 cells through stimulation of SIRT1 activity. RES inhibited NF-κB signaling and protected against microglia-dependent Aβ toxicity (Hu et al., 2016; Maugeri et al., 2018).

RES is also reported to cause AMPK activation and it primarily affects the metabolism including an increase in mitochondrial mass and function such as PGC-1α level, citrate synthase and cytochrome c oxidase complex IV (COXIV) activity. RES administration is further linked with the activation of autophagy and mitophagy processes to enhance the clearance of damaged cells and elevation in FFA recruitment. (Yang et al., 2021). However, AMPK has played a significant role in improving the AD brain pathology via affecting mitochondrial activity as well as neuroinflammation, along with a decline in Aβ content largely by autophagy. (Yang et al., 2021). *In vitro* report also indicated the ameliorative effect of RES against neurodegeneration through regulating mitochondrial dynamics and acting on AMPK-associated pathways within the brain (Gao et al., 2019). Ma et al., 2019 conducted a study by administering RES at a dose of 25 mg/kg in diabetic Wistar rats AD model, which stimulated SIRT1.

A comparative study of RES (5 μM) was performed on two mitochondria-targeted molecules, i.e., SS31 and MitoQ, for various parameters such as mitochondrial biogenesis and mitochondrial fusion/fission. It has been observed that RES is capable of protecting the mitochondria in N2a cells (Manczak et al., 2010). The authors found that RES pretreatment (for 48 h) protected mitochondria of N2a cells in Aβ exposure experimental model. Impaired mitochondrial fusion and fission process induced by Aβ inhibited by RES through modulating the concerned genes' expression including dynamin-related protein 1 (Drp1), mitofusin 2 (Mfn2), mitochondrial fission 1 protein (Fis1) and optic atrophy 1 (Opa1). Levels of PGC-1α protein and increased mitochondrial H_2_O_2_ level induced by Aβ were not modulated by RES alone. Thus RES did not activate the process of mitochondrial biogenesis. But cytochrome c oxidase activity, MPP damage and mitochondrial fragmentation were significantly prevented by RES in cells against Aβ. Therefore, it has been suggested that targeted delivery of RES to mitochondria can exert marked neuroprotective effects against impaired mitochondrial function and dynamics induced by Aβ-associated AD (Cadonic et al., 2016; Islam, 2017).

An *in vitro* study revealed that RES exhibited neuroprotective effect associated with mitophagy by reducing oxidative damage induced by Aβ in PC12 cells. Mitophagy, similar to autophagy, is accountable for the quality control of mitochondria by intracellular reprocessing of impaired mitochondria along with a specific degradation process. Damaged mitochondria promote mitophagy as a result of a decline in mitochondrial membrane potential. Additionally, mitochondrial impairment is associated with oxidative stress, which is known as one of the most contributing factors in AD as well as other various neurological disorders. Therefore, the mechanism of mitophagy can be a potential approach to prevent neuronal oxidative damage by selective elimination of impaired mitochondria. *In vitro* model of AD demonstrated that RES-induced mitophagy plays a protective role against oxidative damage (Komorowska et al., 2020)

Recent studies indicate that developing ND may be modified by autophagy pathways. Pathology of AD is associated with neuronal damaged mitochondria. Autophagy in the mitochondrial membrane is the result of disordered oxidative phosphorylation due to translocation and aggregation of misfolded proteins (Busche and Hyman, 2020; Trinh et al., 2021). As an outcome, neuronal autophagy affects myelination, flexibility of synapses, development of oligodendrocytes and anti-inflammatory action in glial cells (Sindhu et al., 2021; Grover et al., 2022). Ability of autophagy decreases with age leading to Aβ accumulation and liberation of cytochrome c in the membrane of mitochondria, neurodegeneration and apoptosis (He et al., 2017; Kou and Chen, 2017). In addition, atypical autophagic vacuoles are assembled in APP/PS1 AD mouse model (Yang and Zhang, 2020). Reduction in proteolysis of Aβ is due to inequalities amongst autophagy and degeneration (Ahmed et al., 2017). Disability of autophagy in AD increases the expression of PSEN1, thereby increasing the activity of β-secretase leading to increased the production of Aβ (Deng and Mi, 2016; Akter et al., 2021a). RES decreased expression of PSEN1 due to its autophagy-inducing property, suggestive of Aβ synthesis suppression (Islam et al., 2022). RES could decrease the neurodegeneration induced by Aβ by stimulating tyrosyl transfer RNA synthetase-auto-poly-ADP-ribosylation of poly (ADP-ribose) polymerase 1 (PARP1) SIRT1 signaling pathway in PC12 cells, resulting into autophagy (Akter et al., 2021b). RES can be used in the treatment of AD through the stimulation of autophagy. In several studies, the possible neuroprotective outcome of RES in AD has been highlighted (Pourhanifeh et al., 2019; Rahman et al., 2020). RES protects the brain by various mechanisms. Stimulation of appropriate autophagy is an important process followed by RES in AD. The autophagy mechanism of lysosomes is abnormal in AD, which increases the activity of β-secretase, leading to the escalated production of Aβ. It has been concluded by researchers that autophagy signals mediated by RES gives protection in neurodegeneration. Furthermore, these signals thwart a possible reduction in mitochondrial activity (Islam et al., 2022). AMPK, SIRT1 and PGC-1α, all play an important role in modifying the activities of cell by RES (Ahsan et al., 2020; Lu et al., 2021). RES can stimulate SIRT1, enhance the ratio of NAD+/NADH and increase cellular protein clearance influencing CNS ailments via mammalian target of rapamycin (mTOR) reliant or non-reliant approach to improve neurogenesis (Wang et al., 2016). It has been proposed that RES, by activating AMPK, promote the long-life of older people by regulating normal cell autophagy and protecting integrity of mitochondria (Yang et al., 2017).

Results of the study conducted by Wang et al., 2018 revealed that RES treatment caused a significant reduction in Aβ-induced oxidative damage and mitochondrial disorder, as well as a substantial increase in mitophagy. Conversely, 3-MA, an autophagic/mitophagic inhibitor, significantly hampered the protecting effects of RES. This indicates that RES-induced mitophagy provides neuroprotection against apoptosis induced by Aβ through reduction of oxidative stress.

### RES in PD

A past study revealed the ameliorative effect of RES on mitochondrial respiratory function by inducing PGC-1α activity *via* acting on the SIRT1-AMPK pathway (Ferretta et al., 2014). Activation of PGC-1α is known t*o* promote the process of mitochondrial biogenesis followed by improvement in mitochondrial function. Deficiency of ATP molecules and intracellular calcium oscillations are associated to susceptibility of several neuronal sets in PD. ROS, particularly hydrogen peroxide, causes astrocytes calcium signalling due to MAO persuaded dopamine metabolism (Vaarmann et al., 2010). Additionally, a condition of metabolic stress takes place in response to maintaining the calcium homeostasis against the recurrent access of cellular calcium that needs to be compensated by ATP requiring pumps (Gandhi and Abramov, 2012). It has been suggested that open calcium channel (L-type) in neuronal mitochondria is more sensitive to diseases related pathological processes (Surmeier et al., 2011). RES has the potential to interrupt the process of PD development by inhibiting calcium rise (Duncan et al., 2010; Yáñez et al., 2011; McCalley et al., 2014). RES administration exhibited an ameliorative effect on dopaminergic neurons possibly due to modulation of oxidative stress in an experimental model (Alvira et al., 2007; Blanchet et al., 2008). Moreover, a study on RES has determined significant free radical scavenging activity against hydrogen peroxide at a dose level of 100 𝜇g/mL (Konyalioglu et al., 2013). RES plays an important protective role in mitochondrial impairment, chromatin condensation and inflammation, particularly *via* decreasing cyclooxygenase-2 (COX-2) and tumor necrosis factor- α (TNF-α) levels hence, beneficial in neurodegenerative diseases (Jin et al., 2008). Oxidative stress and mitochondrial function are found to be associated with PGC-1α in a transgenic animal model (Mudò et al., 2012). Similar results were obtained by RES treatment as it acted by activating PGC-1α and protecting dopamine against 1-methyl-4-phenyl-1,2,3,6-tetrahydropyridine (MPTP)-induced cell death. *In vitro* activity also demonstrated significant stimulation of PGC-1α by de-acetylating SIRT1 as well as elevating the level of SOD2 and thioredoxin-2 (Trx2) with RES administration. This suggests that RES acts as a neuroprotective agent by following SIRT1/PGC-1α signaling pathway.

SIRT1 has the ability to induce deacetylation and stimulation of heat shock factor 1, in particular, it can disturb the transcriptional process of molecular chaperons as well as heat shock proteins 70 (Hsp70). Hsp70 plays a vital role in the maintenance of cellular protein homeostasis, which primarily prevents the aggregation of abnormal proteins (Raynes et al., 2012; Herskovits and Guarente, 2013). RES reduced the expression of α-synuclein protein in PD associated cellular model via alleviation or partial inhibition of glycogen synthase kinase-3β (GSK-3β) (Simão et al., 2012). Inhibition of GSK-3β provides protection from the damaging effects of oxidative stress on dopaminergic neurons, this suggests that GSK-3β is involved in PD pathogenesis as α-synuclein is produced during phosphorylation of GSK-3β (Ni et al., 2015).

A recent report demonstrated the activation of autophagy by RES *via* modulation of SIRT1/AMPK pathway followed by neuroprotection (Wu et al., 2011). A preclinical study suggested the attenuating effect of RES against drug-related dopaminergic neurodegeneration as well as a behavioral deficit in a rodent model by involving SIRT1 activation (Guo et al., 2016). Hence, RES can act as a potent therapeutic tool against PD because it stimulates SIRT1 followed by microtubule-related protein 1 light chain 3 linked with autophagic degradation of α-synuclein.

Enhanced autophagy is also associated with the inhibition of mitochondrial neuron apoptosis (Lin T.-K. et al., 2014; Park et al., 2016; Kou and Chen, 2017; Limanaqi et al., 2019; Wang et al., 2019). Lin et al., 2014a suggested rotenone, a neurotoxin, prevents autophagic flux and causes apoptosis by stimulating autophagic induction. It showed that RES treatment to SH-SY5Y cells significantly prevented the cell death initiated by rotenone via modulating autophagic induction and overall autophagic flux. Further addition of bafilomycin A1 (an autophagosome-lysosome fusion inhibitor) to the co-treatment group inhibited the RES-induced antiapoptosis effect against rotenone along with acidic vesicular formation, indicating the neuroprotective effect of RES by modulation of autophagy (Lin T.-K. et al., 2014). Though numerous reports are suggesting the mechanism of action of RES behind the process of autophagy induction, still the exact mechanism is not known (Chen et al., 2013; Fu et al., 2014; Park et al., 2016). But dealing with AMPK, Unc-51 like autophagy activating kinase 1 (ULK1) as well as mTOR was considered the most well-established mechanism behind the neuroprotective effect of RES (Chen et al., 2013; Fu et al., 2014; Park et al., 2016; Holczer et al., 2019; Pineda-Ramírez et al., 2020). AMPK is known to regulate cellular homeostasis and stimulation of ULK-1, which leads to acceleration of autophagy by Ser 317 and 777 phosphorylation under the condition of nutrient deficiency or caloric constraint. While in an adequate nutrients state, mTOR promotes inhibition of ULK-1, associated with AMPK, via phosphorylation of Ser 757 (Kim et al., 2011). In a similar condition of caloric restriction, RES can modulate phosphorylation and expression of AMPK (Baur et al., 2006; Dasgupta and Milbrandt, 2007). Maintenance of cell growth, endorsement of anabolic processes along with inhibition of cellular catabolic processes including autophagy are well regulated by mTOR complex I (mTORC1). Additionally, RES shows an ameliorative effect against mTORC1 inhibition induced by autophagy through ATP antagonism by binding with the mTOR ATP-binding site (Park et al., 2016).

Consequently, the role RES in mitochondrial dynamics and biogenesis was also explored in various studies (Ungvari et al., 2011; Peng et al., 2016; Lin et al., 2018; Chuang et al., 2019). In continuation, Lin et al., 2018 revealed the damaging effect of rotenone on mitochondrial fission to modulate impaired cellular material, which was partially attenuated by following the extracellular signal-regulated kinase 1/2 (ERK1/2) signaling pathway, promoting mitochondrial fusion process credited with a reduction in cellular stress along with anatomical improvement in mitochondria.

Elevation in mitofusin 2 expression and regulators of mitochondrial biogenesis including PGC-1α and TFAM with the administration of RES (Chuang et al., 2019). The responsible mechanism for the beneficial effect of RES is possibly through modulation of AMPK signaling pathway because inhibition of AMPK causes a significant reduction in mitochondrial markers (Dasgupta and Milbrandt, 2007).

Additionally, mitophagy is activated by the activation of ULK-1 *via* AMPK pathway and PGC-1α-dependent transcription promotes the process of mitochondrial biogenesis (Mihaylova and Shaw, 2011). The engulfment of injured mitochondria hampers its function via cytochrome c release and cell apoptosis (Wu et al., 2011). A study with RES pre-administration showed a reduction in cytochrome c level, whereas elevation in cellular caspase 3 concentration in mitochondrial dysfunction associated with MPTP or rotenone, leading to a reduction in apoptosis process, which is beneficial in PD treatment (Wu et al., 2011).

In another study, RES was suggested as a neuroprotective agent against H_2_O_2_ or 6-hydroxydopamine (6-OHDA) induced damaging effect by stimulation of SIRT-1 (Albani et al., 2009). As reports have claimed that sirtuin, especially SIRT-1, possesses neuroprotective potential through anti-inflammation, antioxidant and inhibition of apoptosis (Bastianetto et al., 2015).

Ferretta et al., 2014 reported that RES maintained the energy homeostatic condition via modulation of AMPK pathway, SIRT1 and increase in PGC-1α′s targeted genes mRNA expression, which promotes the mitochondrial function, in particular reduction in oxidative stress and elevation in mitochondrial biogenesis. This protective effect is likely to involve activation in citrate synthase events, complex I, basal oxygen demand along with the synthesis of mitochondrial ATP, while alleviation in the amount of lactate, thus directing an oxidative metabolism shift from the glycolytic process.

**FIGURE 3:**
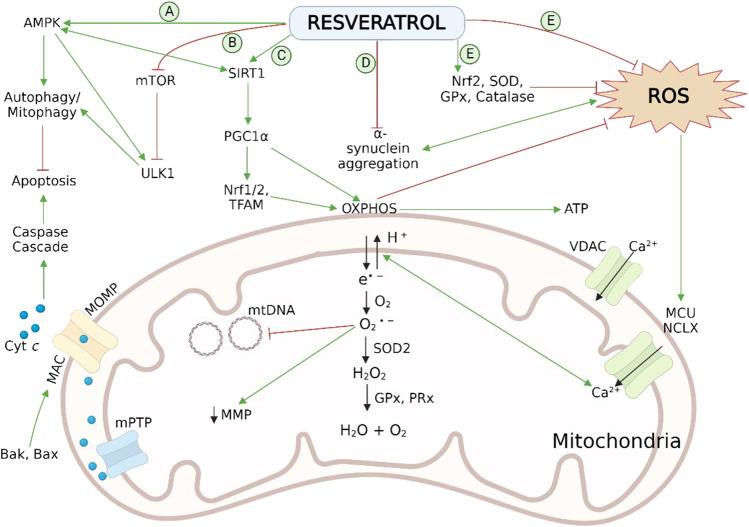
Neuroprotective effect of RES in PD pathogenesis *via* reversal of mitochondrial impairment by **(A)** activation of AMPK and **(B)** inhibition of mTOR and ULK1 leads to autophagy/mitophagy by recruiting autophagosomes followed by inhibition of mitochondrial dependent apoptosis; **(C)** activation of SIRT-1-AMPK signalling pathway enhances the process of mitochondrial biogenesis by persuading PGC-1α, Nrf1/2, and TFAM; **(D)** reduction in aggregation of α-syn aggregation; **(E)** scavenging action on cellular ROS and activation of endogenous antioxidant enzymatic activities. Normal physiology of mitochondrial bioenergetics comprises the mitochondrial OXPHOS system, exist on the inner membrane, which generates ATP to fulfil the cellular energy requirements. During the OXPHOS process, leakage of electrons takes place from mitochondrial complexes I and III, and generates the O2 •− byproducts. These radicals disrupt the Ca^2+^ homeostasis, induces damaging effect on mtDNA and, increases oxidative stress as well as apoptosis associated with mitochondria. Antioxidant mechanisms of mitochondria involves ROS-scavenging enzyme SOD2, GPx and PRx. AMPK, adenosine monophosphate-activated protein kinase; Bak, B cell lymphoma 2 (Bcl-2) homologous antagonist killer; Bax, Bcl-2-associated X protein; cyt c, cytochrome c; GPx, glutathione peroxidase; HO-1, heme oxygenase-1; IMS, intermembrane space; MAC, mitochondrial apoptosis-induced channel; MCU, mitochondrial calcium uniporter; Mn-SOD (SOD2), manganese superoxide dismutase; MOMP, mitochondrial outer membrane permeabilization; mPTP, mitochondrial permeability transition pore; mtDNA, mitochondrial DNA; mTOR, mechanistic (or mammalian) target of rapamycin; NCLX, the mitochondrial Na/Li/Ca exchanger; Nrf1/2, nuclear respiratory factor 1 and 2; O2 •−, superoxide radical; OXPHOS, oxidative phosphorylation; PGC-1α, peroxisome proliferator-activated receptor coactivator-1α; PRx, peroxiredoxins (scavenger and antioxidant) ROS, reactive oxidative species; SIRT-1, sirtuin 1; SOD1/2, superoxide dismutase; TFAM, mitochondrial transcription factor A; ULK1, Unc-51 like kinase 1; VDAC, voltage-dependent anion-selective channel; α-syn, α-synuclein.

Peng et al., 2016 revealed that RES has the potential to reverse the rotenone-induced deficit in PC12 cells by modulating mitochondrial biogenesis. Preadministration of RES at a dose level of 60 μm increased mass of mtDNA and inhibited rotenone-induced decrease of proteins (Drp1, Opa1, Mfn2 and Fis1) associated with the process of mitochondrial fusion and fission. In addition, PGC-1α/TFAM pathway followed by RES activates the mitochondrial biogenesis in rotenone-treated PC12 cell lines, leading to ATP surge along with an antioxidant effect. A preclinical study performed on rats, pretreated with RES *via* i.p. route at 50 mg/kg before rotenone administration also showed improvement in mitochondrial dynamics related to PD (de Oliveira et al., 2016). Therefore, the utilization of RES against damaged mitochondrial dynamics can be a natural and effective approach in PD neurodegenera Figure 3.

### RES in HD

HD is an autosomal and dominantly inherited neurological disease, which is characterized by gradual loss of striatum neurons that affects motor, psychiatric and cognition-related functions and may further cause death (Fu et al., 2012). HD genesis is basically induced by gene mutation involving a repeated sequence of unstable CAG trinucleotide at the N-terminus of the gene encoding Htt (MacDonald et al., 1993). The process of mHtt formation along with polyglutamine generation leads to neuronal dysfunction, cytotoxic aggregates in neurons, and neuronal death in striatum and cortex region (Fu et al., 2012). Therefore, overexpression of Htt fragments in neurons causes HD pathology.

RES is reported to show a neuroprotective effect via SIRT1 stimulation against cytotoxicity induced by polyglutamine mHtt (Feng et al., 2013). Different mechanisms such as oxidative stress, mitochondrial dysfunction and apoptosis are proposed to be responsible for striatal neurodegeneration by triggering mHtt. Activation of p53 is attributed to mediating the damaging effects of mHtt in human neuronal cells. In HD cells and transgenic mice, p53, a tumor suppressor causes dysfunctions and cytotoxicity, therefore, its inhibition prevents the concerned phenotypes (Bae et al., 2005). RES modulates SIRT1 activity defected by mHtt as well as inhibits p53, which is deacetylated and interacted by SIRT1 (Luo et al., 2001; Vaziri et al., 2001). The p53 deacetylation hampers its function and prevents apoptosis relying on p53. Activation of p53 in HD leads to an increase in mitochondrial oxidation (Zhou et al., 2003; Matoba et al., 2006), whereas SIRT1 stimulation due to RES administration makes the cell acclimatize to the situation of energy stress. Due to the potent antioxidant effect, RES can efficiently interrupt mitochondrial oxidation and by modulating SIRT1-PGC1α signaling pathway, it reverses the mitochondrial deficit (Granzotto et al., 2012; Choi et al., 2013; Desquiret-Dumas et al., 2013).

SIRT1 is a nicotinamide adenine dinucleotide (NAD+) associated lysine deacetylase which maintains mitochondrial endurance along with elevated metabolism. SIRT1 activation and its inhibition have been reported to improve HD-related neuropathology.

Naia et al., 2017 explored that RES improves mitochondrial function in both models, i.e., *in vitro* and *in vivo* models associated with HD. RES was administered at two dose levels (1 and 5 μM for 96 h) and observed different effects according to the individual cell type. For instance, 5 μM of RES reinstated matrix metalloproteinase (MMP) level in cortical neurons of YAC128, whereas improvement in striatal neurons MMP was observed at 1 or 5 μM in YAC128. *In vitro* studies show that RES did not stimulate SIRT1. RES exhibited an ameliorative effect against impaired motor and learning function, and increased ETC gene expression encoded with mitochondria in YAC128 mice without affecting the expression of cytochrome c when provided at 1 mg/kg/day by subcutaneous route for 28 days.

Interestingly, RES caused the activation of SIRT1 in the striatum region in both types of mice, i.e., wild as well as YAC128, but in the case of the cortex, no effect on SIRT1 was found in YAC128 mice. Consequently, among other factors, the effect of RES depends on the mice’s brain area. Further work would be required to assess RES potential for enhancing the mitochondrial mass in such brain areas.

### RES in ALS

Past *in vivo* studies showed that RES promoted mitochondrial biogenesis against energetic strain. Daily administration of RES at a dose of 160 mg/kg for 4 weeks reversed the elevated levels of complexes I–V in the lumbar spinal cord’s ventral part in SOD1G93A mice model of ALS. Moreover, triggered mitochondrial fission was observed via an increase in Fis1 expression without modulating Mfn2 level as well as mitochondrial fusion in SOD1G93A mice. Mitochondrial biogenesis induced by RES indicates an increase in phospho-AMPK concentration. RES regulated LC3II and Beclin 1 levels by restoring autophagic flux in SOD1G93A experimental mice. Neurodegeneration induced by mitochondrial dysfunction is associated with ALS (Moreno-Ortega et al., 2016; Bozzo et al., 2017; Carrì et al., 2017) and utilization of mitochondrial protective agents like RES can be a favorable approach for restoring the function and dynamics of mitochondria resulting in improvements in different cellular aspects such as bioenergetic and redox status in patient with ALS.

## Unmet challenges and future prospects

### Positron emission tomography imaging

Continuous rise in ND epidemics requires the development of a new treatment assured by understanding the choice and target along with optimization for *in vivo* authentication. PET neuroimaging technique is a subtle molecular technique, which is sensitive toward brain molecular changes and detects alterations even before their occurrence at structural level. It is a significantly beneficial tool for therapeutic estimation as well as optimization associated with ND, which aids in both research and clinical care. On the basis of PET imaging, antiamyloid approach including c-secretase, passive immunization and β-secretase inhibitors for AD management have entered the clinical phase. It is very difficult to estimate the bio-efficacy of a therapeutic agent on a predicted central nervous system target without any substitute biomarker to understand the consequences of the therapeutic trial. Clinical symptoms evaluation is not an effective way for ND associated therapy analysis as it may cause overlapping of crucial symptoms of different ND. To address these concerns, PET imaging technique delivers the information about localization of metabolic alterations along with quantification of drug distribution. PET scan has been proved to be a beneficial tool in the determination of drug effectiveness. It also provided an insight into the pathophysiological pathway concerned with ND. Recently, the progression of PET radioligands subjected to accurate *in vivo* neuroimaging has been the main research attraction and due to PET, various pathophysiological processes associated with ND (neurotransmission, neuroinflammation and aggregation of protein) could be explored (Dupont et al., 2018).

Terada et al., 2022 utilized PET with [^18^F]2-tert-butyl-4-chloro-5–2H-pyridazin-3-one (BCPP-EF) to measure mitochondrial complex I (site of ROS production) in patients with mild AD. The report suggested that PET with [^18^F]BCPP-EF could efficiently forecast future neurodegeneration by estimation of mitochondrial complex I.

It has been reported that oxidative stress caused by overproduction of ROS in response to mitochondrial respiratory chain damage can be easily imaged by PET with ^62^Cu-diacetyl-bis(N4-methylthiosemicarbazone) (ATSM) technique. This method suggested an increased level of oxidative stress involved in patient with brain-related diseases such as PD, ALS and mitochondrial disease. Level of oxidative stress is the indicator of disease severity and oxidative stress caused by mitochondrial dysfunction that promotes neurodegeneration in a patient (Ikawa et al., 2020).

To date, according to literature reports, there is a lack of data related to PET neuroimaging studies with RES treatment in different ND. PET could be a valuable tool for therapeutic assessment, distribution pattern and mechanism of action determination for the assessment of clinical efficacy of RES.

### Conclusive clinical trial

RES is observed as safe and well tolerated at physiological doses. It showed no toxic effects in human beings upon chronic administration. However, at higher doses, it caused GI discomfort and diarrhea. The neurodegenerative ameliorating effect of RES has been observed at cellular levels and in animal models, which gave investigation grounds for the clinical trial. Still, the translational generalization of outcomes from animal studies could not be achieved. So far, there is a lack of evidence that proves the neuroprotective effect of RES in the human model, likely due to the difference in RES metabolism in rodents and human beings. Small sample size as well as short administration period could be the major limitation in RES’ published clinical trial associated with ND. Therefore, the experimental regime should start long before the clinical symptom occurrence. Early diagnosis of ND with peripheral biomarkers would be an essential requirement for possible long-term trials of RES (Lange and Li, 2018).

There is a requirement for a correlation between brain bioavailability and *in vivo* therapeutic effect to reach any conclusive remark on clinical efficacy of RES against ND. This needs further investigation in human clinical trials.

### Development of an effective drug delivery system

Despite having great therapeutic potential, the RES administration finds several limitations. Important limiting factors of RES are instability, photosensitivity, low solubility and poor ability to permeate through blood brain barrier, and low bioavailability due to short half-life and quick metabolism (Ramalho and Pereira, 2016; Andrade et al., 2018). Additionally, the issue of low bioavailability hinders the required accumulation of drug concentration in targeted tissue needed for successful therapy (Berman et al., 2017). RES is reported to be rapidly metabolized in the liver and is quickly eliminated from the body after absorption. Since drug bioavailability is an important factor for the attainment of a potential therapy against ND, new therapeutic approaches are required to be developed (Ghersi-Egea et al., 2009). Over recent years, nanotechnology has been demonstrated as a promising way for targeted delivery of RES to improve its therapeutic effectiveness (Summerlin et al., 2015). Researchers have improved RES bioavailability via nanoencapsulation led to an increase in its water solubility and prevention of degradation. Nanoencapsulation also aided the reduction of RES-associated toxic effects in healthy tissues (Amri et al., 2012; Arora and Jaglan, 2017). Various nanomaterials have been reported for targeted drug delivery of RES (Peres et al., 2012; Khan et al., 2015; Ramalho and Pereira, 2016).

RES-loaded polysorbate 80-coated polylactic acid nanoparticles with a mean size of 200 nm were reported to show neuroprotective effects against 1-methyl-4-phenyl-1,2,3,6-tetrahydropyridine (MPTP) mediated neurochemical and behavioral changes in C57BL/6 mice model of PD as compared to free RES administration (da Rocha Lindner et al., 2015). RES-loaded solid lipid nanoparticles prepared with apolipoprotein E displayed a spherical shape and were reported to be stable for up to 6 months. It showed *in vitro* cytotoxic effects with no significant toxicity in the hCMEC/D3 cell line. A double-fold increase in permeability through monolayers of hCMEC/D3 was observed in apolipoprotein E functionalized RES-loaded nanoparticles in comparison to untargeted solid lipid nanoparticles (Neves et al., 2016). Low-density lipoprotein receptor ligand peptides conjugated with different mass ratios of polylactic acid-coated mesoporous silica nanoparticles were synthesized. It increased transcytosis through blood–brain barrier, effectively targeted the delivery of RES in the central nervous system, and reduced oxidative stress (Shen et al., 2018). Another research revealed that size reduction of RES in nano-range resulted in an increase in RES permeability across blood-brain barrier with enhanced stability as well as therapeutic effect. Solid lipid nanoparticles of RES functionalized with monoclonal antibodies against transferrin receptor has the ability to act as a promising agent for the treatment of AD. These antibody-conjugated nanoparticles increased the cellular uptake and transportation of RES extract to the brain in AD (Loureiro et al., 2017). Frozza et al., 2010 synthesized lipid-core nanocapsules by using capric/caprylic triglyceride and sorbitan monostearate via technique of interfacial deposition. These nanocapsules showed neuroprotective effect by reducing damaging effects induced by Aβ deposition. The improved efficiency of RES-loaded nanocapsules is likely due to an increase in the concentration of RES within brain tissue (Frozza et al., 2013a; 2013b). Polymeric micelles composed of poly (caprolactone) loaded with RES and coated with polyethylene glycol were prepared. Neuroprotective effects of loaded-RES, as well as free-RES, on viability of PC12 cells were evaluated. Loaded-RES was found to be non-toxic, reduced oxidative stress and caspase-3 activity attributed to enhanced PC12 cells protection against Aβ compared to free-RES (Lu et al., 2009). Wang et al., 2011 developed encapsulated liposomes of RES for protection from PD induced by 6-hydroxy dopamine in rats. It exhibited beneficial effects by significant improvement in rotational behavior, reduction in ROS level, loss of the nigral cells and apoptosis. Palle and Neerati, 2018 explored RES encapsulated nanoparticles’ effect in PD induced by rotenone. RES nanoparticles were developed by antisolvent precipitation in a temperature-controlled manner and stabilized with hydroxypropyl methylcellulose. RES nanoparticles exhibited enhanced bioavailability and therapeutic activity in rats.

## Conclusion

Over recent years, it has been revealed that mitochondrial dysfunction is the key factor responsible for the onset and further progression of different ND. Still, it is not clear if such an incidence is the main causative factor or consequence of neurodegeneration. Besides safety and efficacy, the potential compound should have the ability to modulate mitochondrial function and dynamics to develop a new therapeutic means to treat ND. RES acts on various targets against neurodegeneration, representing it as a remarkable tool for neurological deficit treatment. Along with antioxidant and antiinflammatory properties, RES shows a protective role in the improvement of mitochondrial functions and the process of biogenesis by SIRT1/AMPK/PGC-1α signaling pathway. However, RES has a notable problem of low water solubility. It is right to remember the potential problems related to the possible therapeutic use of RES.
